# Fabrication and in vivo testing of a sub-mm duckbill valve for hydrocephalus treatment

**DOI:** 10.1038/s41378-024-00829-8

**Published:** 2024-12-14

**Authors:** Yuna Jung, Daniel Gulick, Jennifer Blain Christen

**Affiliations:** Department of Electrical, Computer and Energy Engineering, Arizona State University, 650 E. Tyler Mall, Tempe, AZ USA

**Keywords:** Engineering, Microfluidics

## Abstract

Hydrocephalus is characterized by the accumulation of excess cerebrospinal fluid (CSF) in the cranium due to an imbalance between production and absorption of CSF. The standard treatment involves the implantation of a shunt to divert excess CSF into the peritoneal cavity, but these shunts exhibit high failure rates over time. In pursuit of improved reliability and performance, this study proposes a miniaturized valve designed to mimic the natural one-way valve function of the arachnoid granulations and thereby replace the shunts. A benchtop testing setup was employed to characterize the behavior of the fabricated valve. Additionally, an animal study was conducted to assess the valve’s in vivo performance. This involved the injection of saline into the lateral ventricle to elevate intracranial pressure (ICP), followed by the drainage of the saline through the valve inserted into the cisterna magna (CM) to reduce pressure. Our prototype features a silicone duckbill valve design combined with a silicone tube as an inlet. Through benchtop testing, the valve exhibited unidirectional flow with negligible reverse leakage, revealing that critical parameters such as the width of the fluid channel (W) and bill length (L) could be controlled to optimize valve performance. Notably, the valve configuration with W= 0.8mm and L < 0.5mm achieved the lowest cracking pressure (2.22 ± 0.07 mmHg) and outflow resistance (22.00 ± 0.70 mmHg/mL/min) within the low cracking pressure range of conventional shunts. Our observations of the in vivo test demonstrated that when untreated states, pressure differences from baseline to peak exceeded 20 mmHg due to the absence of drainage, resulting in sustained pressure elevation. Conversely, upon treating states by removing the clamp, pressure differences from baseline to peak remained below 5 mmHg, indicating effective drainage of injected saline through the valve. These promising results highlight the potential of the miniaturized duckbill valve as an alternative for ICP management in hydrocephalus, offering improved control and reliability compared to conventional shunting systems. Further research is required to evaluate the valve’s performance as a chronic implant.

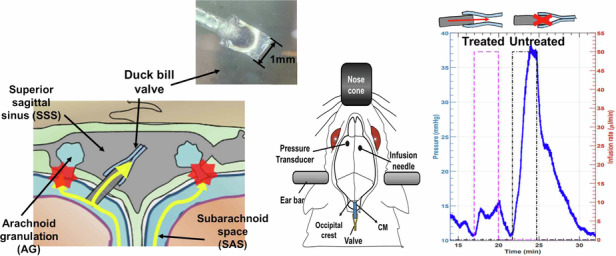

## Introduction

Hydrocephalus, a complex pathological condition with diverse manifestations, arises from an intricate interplay between the production and absorption of cerebrospinal fluid (CSF), ultimately leading to CSF accumulation and subsequent increase of intracranial pressure. This condition, marked by distension within the brain’s ventricular system, is the consequence of obstructions that disrupt the natural flow of CSF or its effective absorption back into the systemic circulation^1–3^. These obstructions can stem from a variety of factors, including congenital malformations, tumors, or inflammatory responses. Depending on the specific location of the obstruction, hydrocephalus is categorized as either communicating or non-communicating. Non-communicating hydrocephalus involves a blockage in CSF flow before reaching the subarachnoid space (SAS), such as a blockage of the aqueducts between ventricles. In cases of communicating hydrocephalus, the CSF can flow all the way to the cortical SAS but the re-absorption of CSF encounters hindrance. This is often attributed to the functional impairment of arachnoid granulations (AGs), which function as one-way valves allowing CSF drainage from under the SAS into the superior sagittal sinus (SSS). These AGs are crucial in facilitating the transport of CSF from the SAS to the venous system, and their dysfunction in this context contributes to the improper drainage of CSF, thereby exacerbating the condition’s effects^4–7^.

The most prevalent and widely used treatment approach for managing hydrocephalus involves the implantation of shunting systems. These systems are designed to divert excess CSF away from the brain to an alternative re-absorption site, thus alleviating intracranial pressure. The core components of a conventional shunt include a proximal catheter, distal tubing, and a valve, each serving a crucial role in regulating CSF flow^8^. While shunting technology has been crucial in managing this condition, its limitations are increasingly apparent, with complication rates, including shunt failures, remaining unacceptably high. Shunt failure rates have been estimated at approximately 15–45% after initial shunt placement, notably, pediatric patients are particularly vulnerable, with alarming failure rates of over 70%, requiring shunt revisions within 10 years^9–13^. Shunt failure, often attributed to obstruction, infection, over- or under- drainage, presents a formidable obstacle in the management of hydrocephalus^14–16^. However, despite decades of refinement in valve design and materials, shunt-related complications persist, challenging the efficacy of this treatment modality^17^. The most frequent cause of shunt malfunction is occlusion of the proximal catheter which can be caused by choroid plexus or ventricular debris^16,18–20^. This limitation emphasizes the need for alternative approaches to address hydrocephalus more effectively.

In response to the need for an alternative CSF drainage mechanism in cases of communicating hydrocephalus, which can circumvent obstructed AGs and establish an internal CSF pathway, this study explores the concept of a synthetic AG, which can be called “artificial AG (aAG)”, implanted within the cranial cavity. This approach aims to replicate the function of AGs as independent valves, enabling direct CSF drainage into the venous sinus. By confining the drainage process within the cranial space, and emulating the AGs’ function, we anticipate a substantial reduction in complications associated with traditional shunt systems. This approach eliminates the use of catheters placed in the subcutaneous space, thereby mitigating a significant portion of complications related to catheter use, including infections, blockages, and breakages. Achieving this goal involves the integration of a miniaturized passive check valve, which resides within the cranium and efficiently directs CSF drainage from the SAS into the SSS, rebuilding a natural drainage system that closely mimicking AGs. Several design iterations have been explored in the pursuit of miniaturized valves as potential replacements for conventional shunting systems for communicating hydrocephalus treatment^21–24^. Oh et al. proposed a 3D dome-petal-shaped microvalve composed of polydimethylsiloxane (PDMS)/Parylene. The cross-cut on the dome acts as a one-way valve based on pressure differences^21^. Lee et al. developed a 3D printed microelectromechanical system (MEMS)-based valve, constructed from hydrogel, to regulate CSF flow by utilizing the hydrogel’s swelling properties^22^. More broadly, other microvalves that were not originally designed for the treatment of hydrocephalus include mechanisms that bear a resemblance to valves used in hydrocephalus treatment^25–29^. Representatively, Park et al. introduced a novel polymeric micro-check valve for a glaucoma drainage device, consisting of three layers, with an intermediate layer featuring a thin valve membrane resting on a valve seat, designed to move in response to pressure differences^25^. Siewert et al. presented a valve with a tonguelike shape, positioned at the inlet area within the drainage tube wall. This flap-like valve mechanism helps prevent stiction, a condition caused by direct contact between the membrane and the valve seat^26^. While these valve designs have demonstrated promising performance, challenges related to reproducibility and long-term durability remain significant areas of ongoing research.

**Fig. 1:**
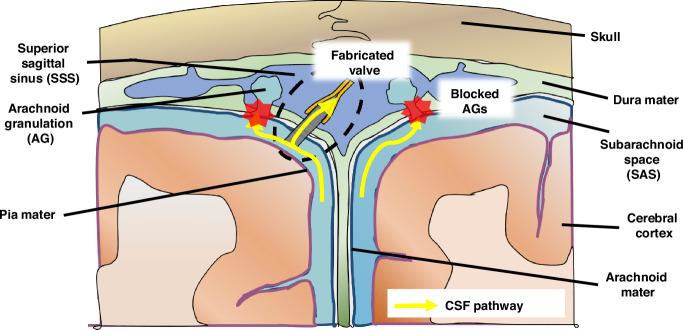
Illustration of alternative methods for CSF drainage in the treatment of hydrocephalus when arachnoid granulations (AGs) fail to regulate CSF flow. The fabricated PDMS duckbill valve can be surgically implanted directly into the dura mater and facilitate CSF drainage from the subarachnoid space (SAS) into the superior sagittal sinus (SSS)

In this paper, we introduce a PDMS duckbill valve as an alternative to conventional shunts for managing CSF dynamics. This valve is designed to facilitate the controlled release of excess CSF from the intracranial space when ICP surpasses a predetermined threshold, known as the cracking pressure (*P*_*T*_). Notably, this duckbill valve can be entirely implanted within the cranium, serving as an aAG to drain CSF from the SAS to the SSS as shown in Fig. 1. What sets our valve apart is its straightforward design, which allows for precise control of the cracking pressure by adjusting key parameters such as thickness, width, and length. This simplicity not only makes fabrication more efficient but also enhances the valve’s customizability for individual patients.

Moreover, the inherent simplicity of the duckbill valve’s design positions it favorably for scaled manufacturing, leveraging established techniques for miniaturization and ensuring high reproducibility.

## Materials and Method

### Fabrication of duckbill valve

**Fig. 2:**
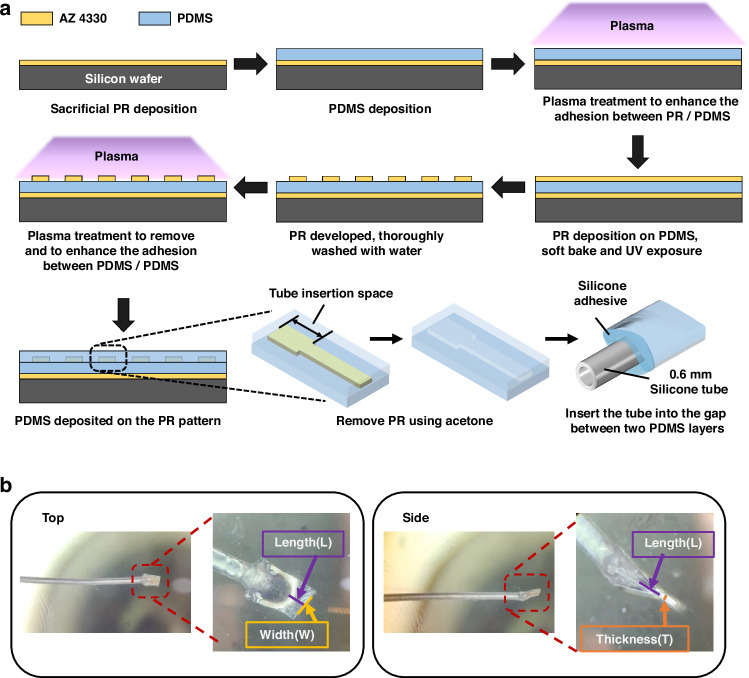
Fabrication and outcome of the duckbill valve. **a** Schematic illustration of the fabrication process. The valve consists of two layers of PDMS, with photolithography used to pattern the fluid channel which remains after the photoresist is removed with acetone. The silicone tube is inserted through this gap until it reaches the end of the tube insertion space. Silicone adhesive is applied around the tube insertion point. **b** Photographs of the fabricated PDMS duckbill valve with silicone tube. On the left, the top view of the valve displays the bill length (L) and fluid channel width (W). On the right, the side view of the valve shows the bill length (L) and thickness (T)

The fabrication process of the PDMS duckbill valve is detailed in Fig. 2a. Initially, a sacrificial layer of photoresist (PR) (AZ P4330) with a thickness of 3–4 *µ*m was spin coated onto a 4 inch silicon wafer (3000 rpm, 30 s). This PR layer facilitates the later removal of PDMS devices from the silicon substrate. Next, a liquid PDMS prepolymer (10:1) (Sylgard 184, Dow) was poured onto the PR-coated substrate, spin-coated at 1000 rpm for 60 seconds, and cured at 70 °C for 2 hours. Subsequently, oxygen plasma etching (PlasmaTherm 790 RIE – Fluorine) was employed for 10 min to treat the PDMS surface. This treatment is essential for enhancing the adhesion between PDMS and a subsequent layer of PR, as inadequate adhesion could lead to the formation of cracks or bubbles on the PR surface^30^. Following the plasma treatment, a 4–5 *µ*m thick layer of PR (AZ P4330) was spin-coated onto the PDMS layer (2000 rpm, 30 s). The substrate was then placed on a hot plate for a soft bake. Achieving a uniform PR layer on the PDMS surface critically depends on the soft bake temperature and duration^31^. Therefore, the soft bake temperature must be gradually increased to 80 °C with 20-30 min to allow sufficient solvent evaporation before UV exposure. Note that abrupt temperature changes can result in cracks on the PR surface. An EVG 620 aligner was used to align the substrate and the photomask to pattern the photoresist on the PDMS surface. In this case, positive PR was used so only the exposed area remained on the surface after developing. After UV exposure, the PR layer was developed using a TMAH-based developer (Microchem Corporation) for 2–3 min. The patterned substrate was then rinsed with water. Before depositing another layer of PDMS onto the PR-patterned PDMS, another round of plasma treatment was applied for 2 minutes to remove any remaining PR to enhance the PDMS-PDMS bond. Care should be taken not to exceed the 2-minute duration for plasma treatment, as prolonged exposure can degrade the PR pattern. A second layer of liquid PDMS (10:1) was then spin-coated onto the substrate. Once completely cured, the PDMS was cut into a small slab. The device was lifted off the wafer, and its edges were trimmed to be slightly wider than the fluid flow channel. The PR pattern, which was sandwiched between the two PDMS layers, was dissolved by flowing acetone through the channel. To complete the fabrication, 0.6 mm diameter silicone tubing was inserted into the gap between the PDMS layers, and the junction between the PDMS and the tubing was sealed with thixotropic silicone adhesive NuSil MED3-4013 (NuSil®, Avantor).

### Benchtop testing

**Fig. 3:**
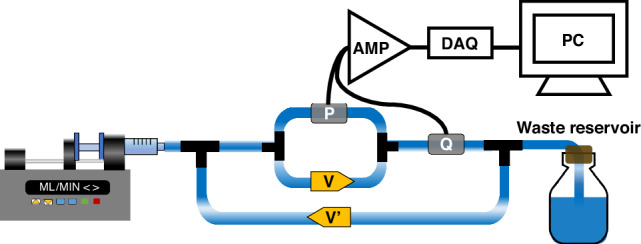
Diagram of the benchtop apparatus used to measure valve behavior while continuously applying both forward and backward directions using a syringe pump. The syringe pump was set to run forward at a rate of 1 ml/min for 60 s and backward at the same rate continuously. To create a closed- loop system that prevents water loss in the syringe, an identical return valve, positioned in the opposite direction of the test valve, was incorporated to enable uninterrupted experimentation. (P: Pressure sensor, Q: flow sensor, V: test valve, V’: return valve)

The experimental setup for evaluating the fabricated valves is depicted in Fig. 3. A programmable syringe pump (Model PHD 2000, Harvard Apparatus) was employed to generate pressure across the valve while recording the resulting output flow. The test valve was positioned in parallel with a pressure sensor (PX26-001DV, Omega) to record its pressure behavior. Simultaneously, the outflow through the valve was measured using a flow sensor (SLI-1000, Sensirion). The outflow was directed toward a waste reservoir. To prevent the one-way flow from causing long-term instability in the measurement of valve performance, a fluid return valve was installed with its inlet placed between the waste reservoir and the flow sensor. During the reverse flow phase, the fluid was split into two pathways: one for the test valve and the other for the return valve. Since the test valve was blocked during reverse flow, fluid could only pass through the return valve. This circulation loop allowed us to observe the valve’s behavior during extended testing periods. To calibrate the pressure sensor, a pressure controller (OB1 MK4, Elveflow) was employed to apply known pressure levels in 5 mbar increments, while measuring the sensor’s output voltage. Similarly, the flow sensor was calibrated using a syringe pump to deliver known flow rates in 0.5 mL/min increments, with simultaneous measurement of the sensor’s output voltage. Data acquisition (DAQ) was carried out using a data acquisition board (NI USB 6216, National Instruments) and SignalExpress software. Before testing, a 10 ml syringe was loaded onto a movable syringe holder. The pump allowed for operation in both forward and reverse directions through the program mode, utilizing profile operations. This feature enabled us to set specific flow rates and time intervals for each direction. By selecting the program mode and configuring the profile operation, we could set the flow rate to 1 ml/min, time interval to 60 s, and pumping direction to forward. Similarly, with the same parameters in the profile operation but with the opposite direction and by selecting the restart operation, the pump performed a repeating sweep between forward and reverse directions. This capability facilitated continuous cycling of the pump, allowing us to assess the check valve’s performance over an extended testing period.

### Animal testing

#### Preparation

All animal experiments were performed in compliance with the protocol (21-1822 R) approved by the Institutional Animal Care and Use Committee (IACUC) at ASU. A total of 7 Sprague Dawley rats were used in the study. In accordance with the protocol, the rats were anesthetized by isoflurane inhalation, and proper anesthesia was confirmed by verifying the absence to a toe pinch reflex. The skin over the surgical area was carefully shaved and cleansed. Lubricant ointment (Puralube®) was gently applied to the eyes to prevent drying. The rat was placed on a heating pad to maintain the body temperature at 37–38 °C. Subsequently, the rat’s head was securely immobilized in a stereotaxic frame using ear bars, and the nose was positioned within a nose cone to deliver a continuous supply of isoflurane/oxygen anesthesia. While it may be beneficial to slightly tilt the nose downward to expose the cisterna magna (CM) more clearly^32^, this adjustment could potentially complicate the precise location of the lateral ventricle relative to bregma. To address this, the rat’s head was oriented in a straight position, firmly secured to the bite bar, and the body was positioned several cm lower than the head height. This arrangement created an approximate 90-degree angle between the head and the upper neck, allowing both CM access and accurate measurement of sterotaxic coordinates for subsequent procedures.

#### Valve insertion

**Fig. 4:**
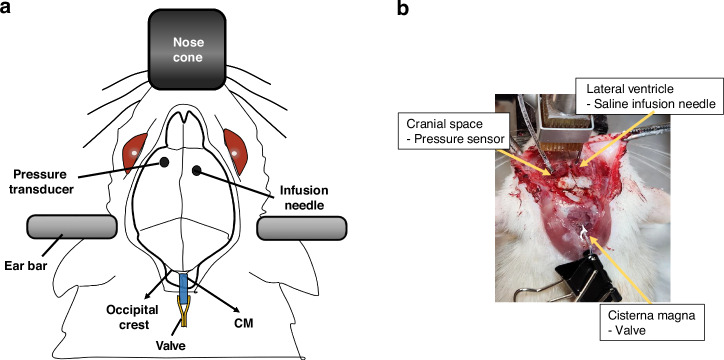
Infusion setup on a rat. **a** Schematic representation of the infusion test setup illustrates the insertion of the infusion needle into the right hemisphere’s lateral ventricle, the pressure transducer into the left hemisphere’s parenchyma, and the valve into the cisterna magna (CM). **b** Photograph of the infusion test setup, displaying the placement of the infusion needle into the right lateral ventricle and the pressure transducer insertion into the left parenchyma. The valve was inserted into the CM and was operated using a clamp to control its treated and untreated state

The valve placement is well shown in Fig. 4a and b in illustration and actual photo, respectively. A midline incision was carefully made in the skin, extending from the occipital crest down to the upper part of the neck using fine scissors. The muscles were gently separated by carefully dissecting the fascia with forceps. Once the fascia was retracted, the superficial muscles were dissected with the same care, revealing an underlying layer of muscles. These deeper muscles were separated along the midline using gentle blunt dissection, revealing the atlanto-occipital membrane. The connective tissue on the membrane was gently handled with cotton swabs to prevent damage. The CM, situated at the base of the skull between the cerebellum and the medulla, presented as an inverted triangle covered by the transparent dural membrane. To aid in orientation, the occipital crest at the posterior end of the skull served as a reference point to access the CM^33^. To facilitate the insertion of the valve inlet at the center of the CM, a 25G needle was used to puncture the CM. The valve inlet (0.6 mm silicone tubing) was inserted through this hole into the CM. A few drops of cyanoacrylate glue were applied to the exposed tubing and the dura around the insertion site, followed by the application of glue accelerator to promptly cure the glue.

#### Infusion setup

The infusion setup is well described in Fig. 4a and b. A single midsagittal incision was made in the skin to expose the surface of the skull. After retracting the skin and fascia, the protective layer on the upper side of the skull was completely removed, and the bone surface was cleansed with saline solution. Two burr holes were carefully created using a high-speed rotary tool with 0.5 mm spherical engraving bit at the appropriate stereotaxic coordinates relative to bregma. For the 25G needle insertion, which was used to inject saline into the right lateral ventricle, stereotaxic guidance was employed. The lateral ventricle was targeted at coordinates of 1.4 mm lateral to bregma, 0.8–1 mm posterior, and 3.8–4 mm deep from the skull surface. The saline injection needle (25G) was connected to a 3 mL BD syringe filled with saline, which was mounted on a syringe pump (Model KDS200, Cole Parmer). Saline was consistently injected at a rate of 50 *µ*l/min, with a total volume of 150 *µ*l, into the lateral ventricle to induce an increase in ICP by introducing excess fluid into the brain. In the opposite hole, located on the left hemisphere, a transducer catheter (Camino® 1104G Catheter Kit – Post Craniotomy Subdural Pressure Monitoring) was inserted to a depth of 4 mm to monitor changes in ICP during the infusion test. After placing the injection needle and transducer, the exposed area was sealed with dental impression material (Defend VPS Impression Material, Light body, Regular set) to prevent any leakage during the test. This material was used instead of dental epoxy to allow easy sensor removal and cleaning^34^. ICP was continuously measured using an ICP monitor (Integra Camino CAM01 Intracranial Pressure Temperature Monitor). The ICP monitor’s output signal was connected to a DAQ device’s analog input channel. SignalExpress was configured to monitor and record the voltage signal continuously.

## Results

### Design

In the development of our duckbill valve, we have adopted a design featuring flexible leaflets constructed from polydimethylsiloxane (PDMS) as shown in Fig. 2. As depicted in Fig. 2a, we have integrated a silicone tube with a 0.6 mm outer diameter between the leaflets to maintain the valve’s inlet side in an open position. A comprehensive illustration of the valve’s mechanism can be found in Fig. 6a. The dynamic operation of this valve relies on its response to pressure differences: when pressure differential exceeds cracking pressure (∆ P > P_*T*_), the leaflets deflect apart, facilitating fluid flow. Conversely, when pressure differential is lower than cracking pressure (∆ P < P_*T*_), it causes the leaflets to close, effectively preventing reverse flow. The valve’s behavior is influenced by a combination of factors, including the internal stresses within the PDMS material resulting from tube insertion and the specific geometric attributes of the leaflets. We have fine-tuned the cracking pressure and outflow resistance by adjusting leaflet parameters, such as the width of the fluid channel (W), bill length (L), and thickness (T). These adjustments allow us to tailor the valve’s performance to meet the requirements of specific applications. For reference, conventional shunts equipped with a differential pressure valve (DPV) are categorized based on their cracking pressure, typically classified as low (3.68 mmHg), medium (7.36 mmHg), or high (11.03 mmHg)^35–37^. The fabricated valve presented here can be manipulated to fit within these specific ranges, providing versatility for individualized applications.

### Valve behavior using benchtop setup

**Fig. 5:**
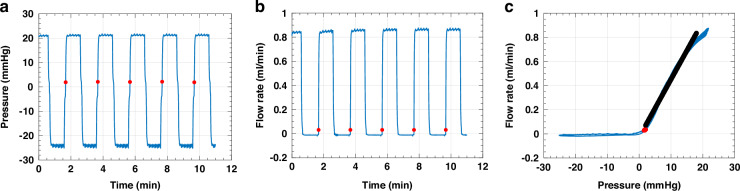
Characteristics of the valve with a 0.8 mm wide and a <0.5 mm length, as determined in the benchtop test. Temporal pressure response (**a**) and flow rate (**b**) of the valve to rectangular-shaped waveforms generated by a syringe pump. **c** Demonstration of the unidirectional behavior of the valve with average reverse flow leakage of −11 ± 0.0006 *µ*L/min. The red dots in **a**–**c** represent the valve’s cracking pressure, determined as the points where the flow rate starts to rise within the range of 20-40 *µ*L/min. Displays outflow resistance (black), calculated using the linear fit function in Matlab, measured across a flow rate of 0.1 to 0.8 ml/min with corresponding pressure ranges. (*n* = 6)

The characteristics of the PDMS duckbill valve were assessed using the benchtop setup depicted in Fig. 3. The temporal response for this valve’s geometry, characterized by a channel width of 0.8 mm, a bill length less than 0.5 mm, and a thickness of 0.1 mm, was studied with regard to its reaction to pressure and flow rate changes during pump operation, as demonstrated in Fig. 5a and 5b with rectangular-shaped waveforms applied via a programmed syringe pump. By analyzing the pressure and flow rate waveforms, we observed that during periods of negative pressure, the flow rate approached zero, indicating no detectable leakage. Upon switching the pump’s direction to forward, the pressure began to rise. Once the pressure reached the cracking pressure (as indicated by red dots), fluid release was initiated by the valve, resulting in an increased flow rate. The valve’s outflow resistance contributed to a continuous pressure increase. When the pump’s direction was reversed, the pressure decreased to negative values, and the flow rate returned to zero. The cracking pressure was defined as the pressure when the flow rate was in the range of 20–40 *µ*L/min. Any flow rates below this range were attributed to environmental artifacts in the setup. These environmental artifacts refer to minor deviations in the flow rate measurements that can arise from various sources in the experimental setup, such as tubing compliance and the pump and sensor response time^38^. The relationship between pressure and flow rate, established through pressure vs. flow rate plotting, is presented in Fig. 5c. The valve exhibited highly directional behavior, demonstrating significant diodicity with averaging reverse flow leakage of −11 ± 0.0006 *µ*L/min. However, this value did not represent the actual reverse flow rate of the valve, which might be considered as leakage. Instead, this can be considered as artifacts due to the environmental factors mentioned above. The measured cracking pressure was 2.22 ± 0.07 mmHg, falling within a low cracking pressure range of standard DPV shunts (3.68 mmHg). Although the pump was set to 1 mL/min, the actual flow rate in our experiments was around 0.83 mL/min (Fig. 5b). This difference in stroke volume was due to resistance and compliance from the tubes and connections in the setup (Fig. 3b). This higher flow rate was necessary to overcome the additional resistance and achieve measurable results. For comparison, the typical CSF formation rate in humans is about 0.3 mL/min. By analyzing the plot, we defined the valve’s outflow resistance (22.00 ± 0.70 mmHg/mL/min) as the average resistance across the pressure range of 2–18 mmHg, utilizing the linear fit function in Matlab.

**Fig. 6:**
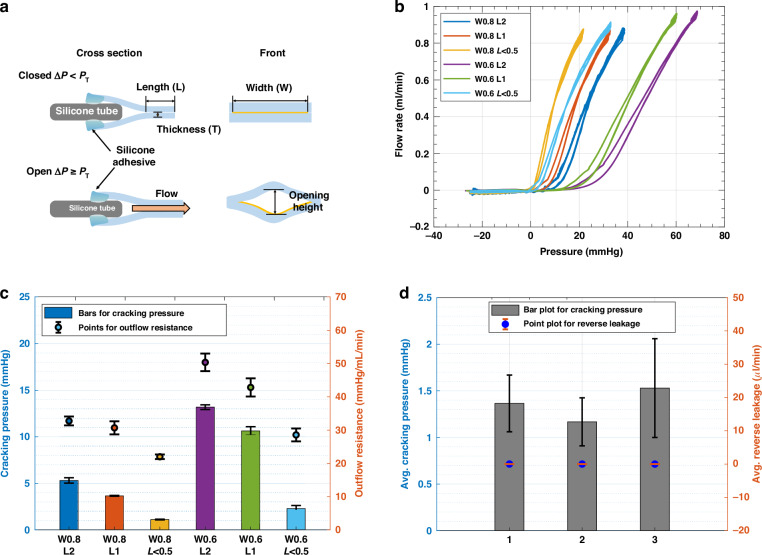
The effect of adjusting valve parameters on the valve behavior. **a** Schematic representation of the valve operation mechanism, illustrating its open and closed states based on the cracking pressure (P_*T*_) in relation to the pressure difference (∆P) between the inlet and outlet sides. **b** Pressure-flow rate relationships to evaluate valve behavior while varying parameters, bill length (L) and fluid channel width (W). **c** Bar and point plots depict the changes in cracking pressure and outflow resistance, respectively, based on the varied parameters. (*n* = 6). **d** Average values of cracking pressure (Bar) and reverse leakage (Point) for three valves during long-term tests over 18,720 cycles

As discussed in the Design section, the valve’s behavior can be controlled by adjusting parameters to achieve target cracking pressure ranges, which may vary on an individual basis for DPV shunts categorized as having high, medium, or low cracking pressure. In Fig. 6a, the thickness (T) of the PDMS layer was kept constant at ≈ 0.1 mm, while we focused on adjusting two key parameters: the width of the fluid channel (W) and the bill length (L). To modify the width, the deposition of PR on the PDMS surface was varied during the fabrication process, leading to alterations in the pattern derived from the photomask. For the adjustment of the bill length, precise trimming was performed under a microscope. The effect of parameter variation on valve behavior was analyzed by examining the relationship between pressure and flow rate, as illustrated in Fig. 6b. A total of six valves was tested for each parameter, showing an average reverse flow leakage of −8 ± 0.0048 *µ*L/min The cracking pressure associated with each parameter setting was presented in Fig. 6c and Table 1. For a W of 0.6 mm, only bill lengths less than 0.5 mm showed cracking pressures within the medium target range for DPV shunts. In the case of a W of 0.8 mm, all bill lengths demonstrated cracking pressures within the target range, offering three pressure range options for DPV shunts: high, medium, or low.

**Table 1:** Cracking pressure of varying parameters

Width	0.6 mm	0.8 mm
Length		
2 mm	25.36 ± 0.05 mmHg	10.62 ± 0.58 mmHg
1 mm	21.25 ± 0.92 mmHg	7.31 ± 0.09 mmHg
*<* 0.5 mm	4.58 ± 0.64 mmHg	2.22 ± 0.07 mmHg

(*n* = 6)

In Fig. 6c and Table 2, we calculated the outflow resistances for each parameter setting using the method of linear fit function in Matlab, as explained earlier. Notably, these outflow resistances are significantly higher than the values observed in DPV shunts, which are typically below 6 mmHg/mL/min. It is important to note that the increased resistances can be attributed, in part, to differences in materials and variations in the experimental setup. In the forthcoming Discussion section, we will explore potential solutions to mitigate and reduce these resistances.

**Table 2:** Outflow resistance of varying parameters

Width	0.6 mm	0.8 mm
Length		
2 mm	50.39 ± 2.63 mmHg/ml/min	30.69 ± 1.35 mmHg/ml/min
1 mm	42.86 ± 2.74 mmHg/ml/min	28.57 ± 1.97 mmHg/ml/min
*<*0.5 mm	32.77 ± 1.94 mmHg/ml/min	22.00 ± 0.70 mmHg/ml/min

(*n* = 6)

To verify the reliability of the valve, we conducted long-term testing with 18,720 cycles (2 min/cycle) of repetitive forward/reverse sequences, controlled by a programmable pump. Three valves were tested, each with a W = 0.8 mm, a L < 0.5 mm, and a T = 0.04 mm. The results are presented in Table 3, which shows the average cracking pressure and average reverse leakage for each valve. While fluctuations in the cracking pressure were observed, these are attributed to unavoidable environmental artifacts related to the test setup. These fluctuations do not indicate degradation of the valve. We examined the valves after the long-term testing and did not observe any degradation. Additionally, during the long-term testing, unlike the short-term testing, we observed bubbles forming inside the tubes. Despite these constraints, the tested valves showed relatively constant cracking pressure and reverse leakage, demonstrating the valve’s reliability and robustness.

**Table 3:** Average values of cracking pressure and reverse flow leakage for 3 valves over 18,720 cycles

Valve	Avg. Cracking Pressure [mmHg]	Avg. Reverse Leakage [µl/min]
1	1.365 ± 0.304	0.0117 ± 0.0041
2	1.167 ± 0.258	0.0017 ± 0.0123
3	1.529 ± 0.53	-0.00412 ± 0.0139

### Animal study

**Fig. 7:**
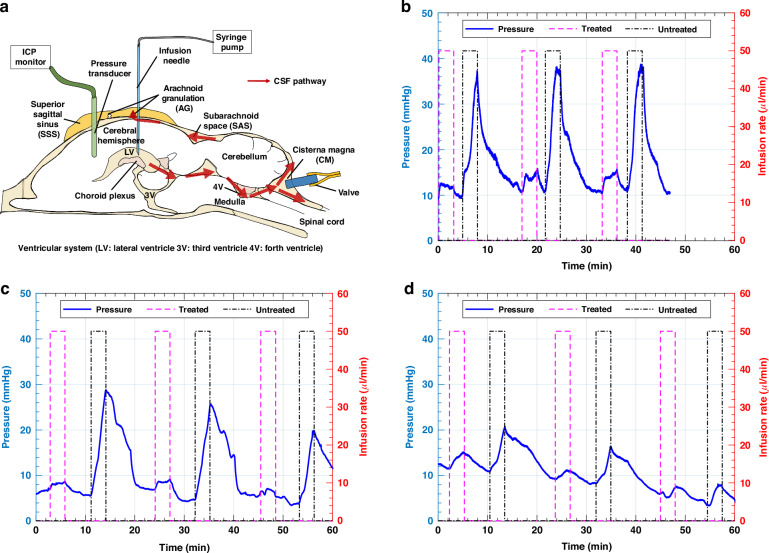
Infusion test on rats. **a** A diagram illustrating the rat brain’s ventricular system and the CSF pathway. (LV: lateral ventricle, 3 V: third ventricle, 4 V: fourth ventricle) Six consecutive waveforms demonstrate three infusions when treated (purple) and three when untreated (black). Each infusion had an injected volume of 0.15 mL at a rate of 50 µL/min. The results of differences in pressure during saline injection of treated and untreated states are shown in **b** Rat 1, **c** Rat 2, and (**d**) Rat 3

The valve (W = 0.8 mm, L < 0.5 mm) was assessed through an infusion test conducted on a live rat to observe its behavior in vivo. Initially, our plan was to implant the valve’s outlet directly into the SSS. However, due to the small size of the rat’s SSS (≈1 mm), implanting the valve without additional support was challenging. Instead, we opted for an alternative location within the CM, which is the SAS situated between the medulla and cerebellum, as shown in Fig. 7a. To understand the CSF pathway, it is essential to note that CSF flows from the lateral ventricle to the CM, then proceeds to the SAS, and finally drains into the SSS through the AGs (Fig. 7a). The choice of the CM insertion offered the advantage of being able to observe the fluid flow through the valve since the valve’s outlet was exposed.

For the in vivo test, we used a total of seven rats. Four rats were successfully tested, as shown in Fig. 7b–d and Fig. S-1. In the other three rats, we encountered inlet blockage issues during the valve insertion into the CM. Despite several attempts to relocate the valve, the CM was damaged, necessitating the use of additional rats. As depicted in Fig. 7b–d, we conducted infusion tests on three individual rats and presented the results of these tests for each rat separately. The results of the infusion tests, summarized in Table 4, show significant differences in ICP changes between treated and untreated states across three cycles for three individual rats. In the treated state, the increases in ICP were consistently lower compared to the untreated state. Additionally, after the injection stopped, ICP returned to the baseline within 2 min for the treated state. The untreated state, the return to baseline was slower (in some cases, the clamp had to be released to facilitate the return to baseline). Figure 7d presented pressure data with baseline shift, which made it difficult to measure the ICP differences accurately. Although this result did not show as considerable changes in pressure compared to the results in Fig. 7b and c, discernible differences between the treated and untreated states were still observable. The consistent reduction in ICP increases in the treated state across all rats indicates the effectiveness of the valve in mitigating the rise in ICP during the infusion tests.

**Table 4:** Summary of ICP Changes in treated and untreated states

Rat	cycle	Condition	Baseline ICP (mmHg)	ICP increase (mmHg)
1	1st	Treated	9.50	3.02
		Untreated	10.04	26.87
	2nd	Treated	10.90	3.52
		Untreated	10.90	26.73
	3rd	Treated	10.50	3.73
		Untreated	11.16	26.75
2	1st	Treated	7.10	1.50
		Untreated	5.70	23.10
	2nd	Treated	6.90	2.27
		Untreated	4.80	21.00
	3rd	Treated	5.42	0.91
		Untreated	3.80	15.95
3	1st	Treated	11.54	3.52
		Untreated	10.82	9.77
	2nd	Treated	9.20	1.78
		Untreated	8.34	7.80
	3rd	Treated	5.96	1.26
		Untreated	3.57	4.34

## Discussion

This study represents progress towards miniaturized valves to replace shunts in managing communicating hydrocephalus. We have successfully developed a miniaturized PDMS duckbill valve with a straightforward design, composed of two leaflets that open and close in response to pressure differentials, using the widely adopted biocompatible material, PDMS. The simplicity of our valve design allows for precise control of its behavior by adjusting structural parameters such as fluid channel width (W), bill length (L), and thickness (T). The measurements confirm that the valve exhibits highly unidirectional behavior, facilitating outflow in the forward direction with negligible reverse flow leakage. Since the experimental setup caused some deviations due to environmental artifacts^39^, such as tubing compliance—slight expansions or contractions of the tubing due to pressure changes—and the pump and sensor response time—the brief period it takes for the syringe pump and the sensor to stabilize the flow rate after changing direction—we recognize the need for improvements. To mitigate these deviations, we plan to enhance the setup by reducing its overall size and using less resistive tubing for connections. By manipulating the parameters, we demonstrated the ability to adjust the cracking pressures of the valve within the targeted range of conventional DPV shunts.

However, it is important to note a significant limitation — the relatively high outflow resistance of the valve compared to DPV shunts. These shunts generally maintain outflow resistances lower than 6 mmHg/mL/min, while our lowest observed resistance, seen in valves with W 0.8 mm and L < 0.5 mm, was 22.00 ± 0.70 mmHg/ml/min. This elevated resistance stems from the diameter of the silicone tube integrated into the valve inlet, which contributes an additional resistance of about 11 mmHg/mL/min as measured from the benchtop using 5 mm length of 0.6 mm outer diameter tubing (data not shown). This diameter was selected for these initial rodent tests. Future tests in large animals may allow wider tubing, with lower resistance. Alternative means to lower the tubing resistance may use low-adhesion coatings such as fluorosilicone or parylene, or may use multiple inlet holes as used in standard ventricular catheters.

This fabricated valve was tested in vivo by placing it in the CM to evaluate its ability in reducing ICP. This was achieved by draining excess fluids that were artificially injected into the lateral ventricle, thereby increasing ICP and initiating flow through the CSF pathway from the lateral ventricle to the CM. The decision to place the valve in the CM was primarily due to anatomical constraints in the rat model. Specifically, the space between the SAS and SSS in rats is too small to accommodate the valve, making it impractical to place it in the same location as would be intended in larger animals or human applications. Thus, the CM was chosen as an alternative site for CSF drainage in the rat experiments.

For conducting an infusion test, it is crucial to consider both the injected volume and the rate of saline injection to minimize potential harm to the rats’ brains. Initially, we attempted an injection volume of 0.2 mL with the rate of 17 *µ*l/min. As shown in Fig. S-1, we conducted six cycles, and this approach took an extended amount of time to return to baseline (≈ 20 - 30 min). The cumulative duration exceeded 3 hours, which may be excessive for rats under anesthesia and could potentially pose a risk to their health^40,41^. Consequently, we decided to increase the infusion rate to 50 *µ*L/min while reducing the volume to 0.15 mL to better suit our experimental setup. After establishing a more reasonable volume and rate, the test was conducted, and as depicted in Fig. 7b–d, substantial pressure differences between the treated and untreated states were observed, confirming its effectiveness in reducing ICP by draining injected saline.

The test shown in Fig. 7d exhibited a smaller pressure difference between the treated and untreated conditions compared to the other two results. This variability in the infusion test results can be attributed to several factors. One primary factor is the rats’ individual physiological and anatomical conditions, including their autoregulatory capacity^42–44^. This capacity involves the brain’s compensatory mechanisms that maintain stable internal pressures despite changes in CSF volumes. Rats with stronger adaptability and more effective compensatory mechanisms may exhibit more stable and lower pressure differences within their brains compared to those with weaker adaptability. Another potential factor contributing to the variability in the results is the insertion of the infusion needle and transducer into the brain. During this procedure, we noted a brief rise in ICP, followed by a return to the normal range (data not shown). This initial perturbation may be attributed to the insertion process, potentially resulting in temporary damage to the rats’ brains. We took great care during the insertion process to ensure the preservation of the CSF pathway’s integrity, particularly when introducing the sensor tip due to its relatively large dimensions (1.35 mm in diameter and 2.5-3 mm in length) compared to the size of the rat’s brain. While inserting the tip into the brain, there existed a risk of unintentional damage to the ventricular system, a crucial component of the CSF pathway. This potential risk could have resulted in the inadvertent leakage of injected saline into other brain regions, deviating from the intended CSF pathway. Such deviations, possibly exacerbated by ventricular collapse, may have contributed to the variability observed in the results. Additionally, sensor drift can affect the overall result. This drift, which involves gradual changes in the sensor’s baseline readings over time, can introduce variability and potentially obscure the true measurements of ICP changes. Furthermore, during the infusion test, we observed that the respiratory rate of the rats increased slightly when the ICP rose to the peak pressure which seemed to be very high. However, the infusion was stopped within one minute, which prevented any serious impact on the rats.

One critical aspect highlighted in our animal study is the need for a supporting tool during valve insertion to minimize the risk of brain damage during surgery. As mentioned earlier, we initially attempted to implant the valve between the SAS and SSS but encountered difficulties due to the complexity of the procedure. The insertion tool is essential for ensuring safe and precise valve placement, especially when working with live animals. Therefore, the development and refinement of suitable insertion tools should be a priority for further research. Regarding future work, we plan to monitor the difference in ICP in the rats’ brains while they are freely moving after surgery. This monitoring is essential because ICP can vary depending on the rat’s body position^45^. To achieve this, we will need to reconstruct the setup using a wireless telemetry system to monitor ICP^34^. Additionally, we need to consider the potential dislocation of the valve. To address this, we plan to develop a grommet that can provide additional stability by securely anchoring the valve within the intracranial area. This makes long-term animal tests possible, allowing us to feasibly observe the valve performance under various physiological conditions, including changes in body position and activity levels. By monitoring these variables, we can gain a more comprehensive understanding of the valve’s behavior in a living system. Moreover, these tests will enable us to predict the possibility of valve failures, such as occlusion, by identifying any potential issues that may arise over time. This approach will provide valuable insights into the durability and reliability of the valve in real scenarios. Additionally, rather than injecting saline directly into the lateral ventricle, we are exploring alternative methods to increase ICP that do not interfere with rats’ mobility. Some methods used in modeling hydrocephalus involve substances like kaolin^46–49^ or silicone oil^50^. Therefore, in our case, where we aim to induce communicating hydrocephalus, the selection of the injection site for these substances becomes a critical consideration that requires further investigation.

## Conclusion

In this study, we have successfully developed a miniaturized PDMS duckbill valve that exhibited unidirectional fluidic dynamic and negligible reverse flow leakage, as confirmed through benchtop testing. By adjusting key parameters, we demonstrated that the valve’s behavior can be precisely controlled to align with the range of cracking pressures typical of conventional DPV. The simplicity of the duckbill valve allows for parametric design within a tractable design space. Our live animal experiments have provided further insights into the valve’s functionality within the physiological context. We observed that the valve operated as expected when inserted into live animals, effectively reducing elevated ICP by draining excess saline when necessary. It is important to note that animal-to-animal variations in the pressure differences between the treated and untreated states were observed, likely due to differences in each rat’s anatomical and physiological conditions. Nonetheless, our results confirmed the valve’s ability to alleviate increased ICP by efficiently draining excess fluid when placed in the CM, a part of the SAS. For future work, we plan to conduct chronic tests in animal models to further validate the valve’s performance when implanted between the SAS and SSS. To achieve this, we will need to develop a supporting tool for safe and precise valve insertion into the brain. Additionally, we will explore alternative methods for inducing communicating hydrocephalus and implement a wireless telemetry system for continuous ICP monitoring before and after valve implantation.

## Supplementary information

Supplemental Material

## Data Availability

Data are available upon request.
