# Supplementary material for: Fabrication and in vivo testing of a sub-mm duckbill valve for hydrocephalus treatment
Source: Microsyst Nanoeng. 2024 Dec 14;10:190. doi: 10.1038/s41378-024-00829-8 (PMC11646279; doi:10.1038/s41378-024-00829-8)
Supplement: Supplementary file 1 — Supplemental Material [file 41378_2024_829_MOESM1_ESM.docx]

**Supplementary information.** In the supplementary material, Fig. S-1 displays the pilot trial of the infusion test.

Fig. S-1: Infusion test was conducted at a rate of 17 *µ*l/min and volume of 0.2 mL. The injections were performed six times; the first three were conducted in a treated (unclamped) state, and the remaining three were conducted in an untreated (clamped) state. Clear pressure differences were observed, with values of 3 – 5 mmHg in treated states and 21 – 25 mmHg in untreated states.

During the test, the resting time (after injection stopped) was set to 20 min, representing the time required to return to the baseline. Despite the specified resting time, the baseline consistently increased throughout the test. Notably, even after releasing the clamp at the end, the pressure did not return to the baseline. This observation suggests that repeated injection of saline in an untreated state may cause significant damage to the rat due to accumulation of the saline. Moreover, an extended duration of the test could potentially harm the rat’s brain. Therefore, to mitigate these concerns, the infusion rate and volume were adjusted and the injections alternated: one was performed in a treated state, followed by another in an untreated state, and so on as shown in Fig. 7.
